# Preparation of Remote Plasma Atomic Layer-Deposited HfO_2_ Thin Films with High Charge Trapping Densities and Their Application in Nonvolatile Memory Devices

**DOI:** 10.3390/nano13111785

**Published:** 2023-06-01

**Authors:** Jae-Hoon Yoo, Won-Ji Park, So-Won Kim, Ga-Ram Lee, Jong-Hwan Kim, Joung-Ho Lee, Sae-Hoon Uhm, Hee-Chul Lee

**Affiliations:** 1 Department of Advanced Materials Engineering, Tech University of Korea, Siheung 15073, Republic of Korea; pullat@tukorea.ac.kr (J.-H.Y.); wonji1221@tukorea.ac.kr (W.-J.P.); swkim9193@tukorea.ac.kr (S.-W.K.); zxc8909891@tukorea.ac.kr (G.-R.L.); rmfladl91@tukorea.ac.kr (J.-H.K.); 2 EN2CORE Technology Inc., Daejeon 18469, Republic of Korea; shuhm@en2core.com; 3 Korea Evaluation Institute of Industrial Technology, Seoul 06152, Republic of Korea; plasma@keit.re.kr

**Keywords:** HfO_2_, plasma-enhanced atomic layer deposition (PEALD), remote plasma, memory window, trapped charge density, plasma damage

## Abstract

Optimization of equipment structure and process conditions is essential to obtain thin films with the required properties, such as film thickness, trapped charge density, leakage current, and memory characteristics, that ensure reliability of the corresponding device. In this study, we fabricated metal–insulator–semiconductor (MIS) structure capacitors using HfO_2_ thin films separately deposited by remote plasma (RP) atomic layer deposition (ALD) and direct-plasma (DP) ALD and determined the optimal process temperature by measuring the leakage current and breakdown strength as functions of process temperature. Additionally, we analyzed the effects of the plasma application method on the charge trapping properties of HfO_2_ thin films and properties of the interface between Si and HfO_2_. Subsequently, we synthesized charge-trapping memory (CTM) devices utilizing the deposited thin films as charge-trapping layers (CTLs) and evaluated their memory properties. The results indicated excellent memory window characteristics of the RP-HfO_2_ MIS capacitors compared to those of the DP-HfO_2_ MIS capacitors. Moreover, the memory characteristics of the RP-HfO_2_ CTM devices were outstanding as compared to those of the DP-HfO_2_ CTM devices. In conclusion, the methodology proposed herein can be useful for future implementations of multiple levels of charge-storage nonvolatile memories or synaptic devices that require many states.

## 1. Introduction

Scaling of the silicon nitride-based charge-trapping layers (CTLs) used in NAND flash memory devices is challenging due to the recent ultra nano-sized processing and high integration of semiconductor devices [1,2,3,4]. With a decrease in the required film thickness, the trapped charge density (*N_t_*) of the CTL decreases, reducing the memory window (∆*V_FB_*) margin that can distinguish the device on/off states at the same operating voltage and program/erase (P/E) time. Additionally, the increase in leakage current owing to the decrease in film thickness degrades the memory retention characteristics, thereby leading to problems in terms of device reliability. To address these issues, researchers have been actively applying high-k materials, such as HfO_2_, Al_2_O_3_, TiO_x_, ZnO, and ZrO_2_, to CTLs [5,6,7,8,9,10]. High-k-based oxides exhibit advantages, including small equivalent oxide thicknesses (EOTs), large band offsets to Si, and high *N_t_* values, over conventional silicon nitride. Moreover, the films of these oxides are expected to achieve the memory characteristics needed for device operation even at a thickness of a few nanometers [11]. Charge trapping properties of high-k-based oxides have been improved by various methods such as noble metal doping [11,12,13], nanocrystallization [14,15,16,17], and high-temperature heat treatment [18,19]; however, these methods have the disadvantage of difficult application to actual mass productions. Furthermore, thin films with charge trapping properties have been achieved by changing the deposition method. Recent studies have reported variations in the charge trapping properties of HfO_2_ thin films with respect to the atomic layer deposition (ALD) temperature and reactant activation [8,20].

ALD is currently the dominant process for depositing thin films with thicknesses of several nanometers. Based on the energy transfer method used for the activation of the reactive gas, ALD is divided into thermal and plasma-enhanced (PE) ALD. Among them, PEALD is mainly used at present due to its lower process temperature, higher film density, faster deposition rate, and shorter one-cycle time [21]. PEALD is classified into direct-plasma (DP) and remote plasma (RP) ALD. However, in the case of DPALD, where the plasma is discharged in the process chamber to deliver energy to the reactive gas, ions in the plasma can bombard the substrate or film surface, causing interfacial damage and degrading film properties [22,23,24]. RPALD, in which the plasma discharge area is separated from the process chamber and only activated radicals are injected into the process chamber, can solve this plasma damage problem. Nevertheless, the lifetimes of plasma-activated radicals are not long; thus, optimization of equipment structure and process conditions is essential to obtain thin films with the required properties [25,26].

In this study, we fabricated metal–insulator–semiconductor (MIS) structure capacitors using HfO_2_ thin films separately deposited by RPALD and DPALD and determined the optimal process temperature by measuring the leakage current and breakdown strength as functions of process temperature. Furthermore, the ∆*V_FB_* characteristics of each capacitor were investigated using capacitance–voltage (C–V) measurements. The effects of the plasma application method on the charge trapping performances of the HfO_2_ thin films and properties of the interface between Si and HfO_2_ were analyzed via electrical analyses. Additionally, the formation of an interfacial layer between Si and HfO_2_ thin films was verified by cross-sectional observation of the device. Finally, we synthesized charge-trapping memory (CTM) devices using the developed interfacial layer as a tunneling oxide (TO), RP- or DP-HfO_2_ thin films as CTLs, and DP-Al_2_O_3_ thin film as a blocking oxide (BO). Then, we examined the applicability of the devices to actual memory devices via electrical measurements of the corresponding properties such as ∆*V_FB_*, P/E speed, memory retention time, and cycling endurance.

## 2. Materials and Methods

### 2.1. Fabrication of Devices

A four-inch p-type (100) Si wafer with a specific resistivity of 1–30 Ω∙cm was washed by SC-1 cleaning and then immersed in buffered oxide etchant for approximately 30 s to remove the native oxide on the Si wafer. HfO_2_ and Al_2_O_3_ thin films were deposited on the resulting wafer using a PEALD system (iOV-dx2, iSAC Research, Daejeon, Republic of Korea). DP was generated by a built-in plasma generator in the PEALD equipment. RP was produced by an RP system (En2ra-RPS, EN2CORE Technology, Daejeon, Republic of Korea) in a separate room from the primary chamber, and the radicals were transferred to the main chamber via a shower head based on pressure difference. Tetrakis(ethylmethylamino)-hafnium (TEMAH, iChems, Hwaseong, Republic of Korea) and trimethylaluminum (TMA, iChems, Hwaseong, Republic of Korea) were employed as precursors for HfO_2_ and Al_2_O_3_ thin-film deposition, respectively, and O_2_ was used as the reactive gas. Subsequently, Pt electrodes with diameters of 200 μm and thicknesses of 50 nm were formed by the lift-off method. Pt deposition was conducted for 3 min at room temperature using a direct current magnetron sputter. Finally, post metallization annealing was performed for 20 min under a N_2_ atmosphere at 400 °C using rapid thermal annealing equipment.

### 2.2. Evaluation of Device Characteristics

Thicknesses of the deposited HfO_2_ and Al_2_O_3_ films were measured using an ellipsometer (Elli-SE, Ellipso Technology, Suwon, Republic of Korea). Cross-sectional morphologies and crystallinities of the films were investigated via field-emission transmission electron microscopy (TEM) (FE-TEM, Tecnai G2 F20, FEI, Hillsboro, OR, USA). Furthermore, compositions and chemical bonding states of the HfO_2_ films were analyzed by X-ray photoelectron spectroscopy (XPS, AXIS-NOVA, Manchester, UK). Electrical characteristics, such as current–voltage (I–V) characteristics, ∆*V_FB_*, and P/E speed, of the device were evaluated using a semiconductor characterization system (4200A-SCS, Keithley, Cleveland, OH, USA) connected to a micro probe station (APX-6B, WIT Co., Suwon, Republic of Korea).

## 3. Results and Discussion

**Figure 1:**
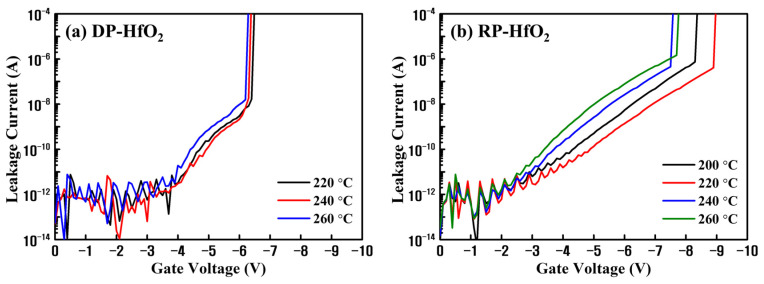
Current–voltage (I–V) characteristics of the HfO_2_ metal–insulator–semiconductor (MIS) capacitors based on the films deposited by (**a**) direct-plasma (DP) atomic layer deposition (ALD) (DPALD) and (**b**) remote plasma (RP) ALD (RPALD) as functions of deposition temperature.

Under the optimized process conditions reported in our previous studies [27,28], MIS capacitors were fabricated by depositing DP- and RP-HfO_2_ thin films on p-Si wafers and developing Pt electrodes. HfO_2_ thin films were deposited by splitting the temperature in the range of 200–260 °C, which was considered the process window, and the thickness of the deposited film was determined to be approximately 10 nm by the ellipsometer. Figure 1 shows the results of the I–V measurements conducted to measure the leakage currents and breakdown strength characteristics of the MIS capacitors based on DP- and RP-HfO_2_ thin films. The current was calculated with an increase in the negative bias. The leakage current and breakdown strength of the DP-HfO_2_ MIS capacitor slightly varied with an increase in the process temperature (Figure 1a). This suggested that the ions bombarded on the thin film by DP further participated in the reaction of the precursor with the reactive gas, mitigating the impact of process temperature on the leakage current and breakdown strength. In contrast, the leakage current and breakdown voltage characteristics of the RP-HfO_2_ MIS capacitor changed with an increase in the process temperature (Figure 1b). The leakage current was lowest, and the breakdown voltage characteristics were highest at 220 °C. This was expected to be owing to the strong influence of thermal energy on the reaction of the radicals activated by RP with the sources adsorbed on the film during film formation. Therefore, determining the optimum process temperature for RPALD of thin films by electrical characterization, for example, I–V measurement, is necessary. The breakdown field of the HfO_2_ MIS capacitor based on the DP-HfO_2_ film (DP-HfO_2_ MIS capacitor) deposited at 220 °C was approximately 3 MV/cm lower than that of the capacitor based on the RP-HfO_2_ film deposited at 220 °C (RP-HfO_2_ MIS capacitor). In the DP method, deposition and plasma discharge occur in the same space, which can damage the substrate and thin film via ion bombardment [22,23,24]. In the current DP process, HfO_2_ thin films were deposited on the Si wafer surface, which was subjected to ion bombardment. Consequently, the formation of unstable interfacial layers and defects within the films was anticipated, leading to a reduction in the breakdown fields of the corresponding capacitors.

**Figure 2:**
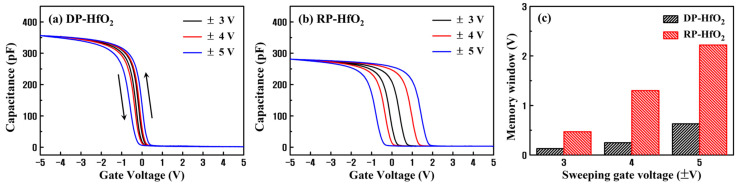
Capacitance–voltage (C–V) characteristics of (**a**) DP- and (**b**) RP-HfO_2_ MIS capacitors as functions of the sweeping voltage and (**c**) the corresponding memory windows.

Prior to the analysis of the interfacial damage and internal defects in thin films, capacitance–voltage (C–V) measurements of DP- and RP-HfO_2_ MIS capacitors were performed in a forward–backward dual sweeping fashion at room temperature and 1 MHz. C–V curves of both DP- and RP-HfO_2_ MIS capacitors demonstrated counterclockwise hystereses (Figure 2a,b, respectively). Counterclockwise hysteresis is a typical hysteresis loop caused by charge trapping. When an electron is trapped at a charge trapping site in an oxide film under a positive bias, the flat band voltage (*V_FB_*) shifts in the positive direction, which is called the program state. However, when a hole is trapped under a negative bias and an electron is detrapped, *V_FB_* shifts in the negative direction, which is called the erase state. ∆*V_FB_* is defined as the difference between the *V_FB_* values in the program and erase states and increases with an increase in the sweeping voltage. For the DP-HfO_2_ MIS capacitor, ∆*V_FB_* slightly increased with an increase in the sweeping voltage. In contrast, for the RP-HfO_2_ MIS capacitor, ∆*V_FB_* significantly increased with an increase in the sweeping voltage, reaching 2.22 V at a sweeping voltage of ±5 V. This indicated excellent charge trapping efficiency and high potential of the RP-HfO_2_ thin film for application as a CTL [7,29]. For the application of oxide thin films as CTLs, the *N_t_* in these films must be high. *N_t_* values per unit areas of DP- and RP-HfO_2_ thin films can be calculated at the point where the ∆*V_FB_* is saturated with an increase in the sweeping voltage using the following equation [18,30]:

(1) $$N_{t}=\frac{C_{ox}∆V_{FB}}{qA}$$

where *C_ox_* is the capacitance in the accumulation region, *q* is the charge of the electron, and *A* is the effective area of the Pt top electrode. Using Equation (1), we calculated the *N_t_* values of the DP- and RP-HfO_2_ thin films at the sweeping voltage of ±5 V; the *N_t_* values were 4.48 × 10^12^ and 1.25 × 10^13^ cm^−2^, respectively. The *N_t_* of the RP-HfO_2_ thin film were more than twice that of the DP-HfO_2_ thin film. However, these values were evaluated before the saturation of the ∆*V_FB_* values of the capacitors by the sweeping voltage. The maximum *N_t_* for each film could not be calculated because the film broke down before the saturation of ∆*V_FB_*. The center of hysteresis for the DP-HfO_2_ film is shifted towards negative voltage, whereas the center of hysteresis for the RP film is shifted towards positive voltage. This opposing shift can be attributed to their distinct distributions of oxide traps, which will be elaborated upon in the results of constant current stress measurements as below.

**Figure 3:**
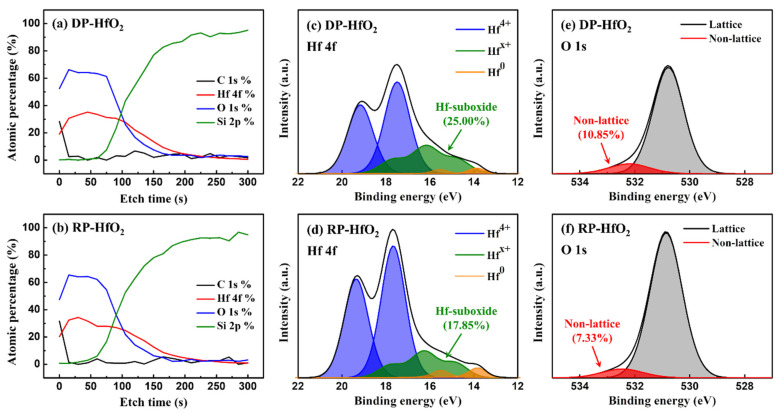
Comparison of (**a**,**b**) X-ray photoelectron spectroscopy (XPS) depth profiling, and (**c**,**d**) Hf 4f and (**e**,**f**) O 1s narrow scan XPS patterns of DP- and RP-HfO_2_ thin films.

Charges are trapped in high-k oxide thin films by the intrinsic defects, such as O vacancies and interstitial O atoms, in the films [31]. Therefore, we indirectly compared the *N_t_* values of the DP- and RP-HfO_2_ thin films by measuring the ratio of the number of lattice bonds to the number of non-lattice bonds in the film via XPS. XPS depth profiling demonstrated that the C 1s atomic percentages in both DP- and RP-HfO_2_ thin films were negligible, except for those on the surfaces (Figure 3a,b, respectively). The presence of C in the thin films is attributed to the incomplete reaction of the reactive gas with the precursor containing C. Thus, both films were deposited under optimal process conditions. Hf 4f and O 1s narrow scans were conducted on the bulk portion of each thin film. Each peak was analyzed using CasaXPS (Version 2.3.25PR1.0). At first, the Shirley-type background was removed from all spectra, and the peaks were fitted using a Gaussian–Lorentzian function. For the deconvolution of the Hf 4f peaks in Figure 3c,d, the ratio of Hf 4f_5/2_ peaks to Hf 4f_7/2_ peaks was fixed at 3:4 [32]. In this case, the spin-orbit splitting was 1.66 eV. We also discovered that the sub-oxide Hf^x+^ peak was represented by the sum of two doublets, and the metallic Hf^0^ peak was in agreement with the data reported in the literature, that is, it appeared at a distance of approximately 3.4–4.1 eV from the Hf^4+^ doublet [33,34]. After peak deconvolution, the percentages of non-stoichiometric HfO_2−x_ in DP- and RP-HfO_2_ thin films were 25.00 and 17.85%, respectively. The O 1s peak can be deconvolved into a lattice peak owing to O bonding in the full crystal and a non-lattice peak due to bonding of O vacancies, O-H, and C-O (Figure 3e,f). In this case, the non-lattice peak emerges at a distance of approximately 1.4–1.6 eV from the lattice oxide peak [35]. The percentages of the non-lattice peaks for DP- and RP-HfO_2_ thin films were evaluated to be 10.85 and 7.33%, respectively. Higher amounts of non-stoichiometric hafnia and non-lattice oxygen can indicate more intrinsic defects. According to the results of Hf 4f and O 1s XPS, we can infer that the number of intrinsic defects in the DP-HfO_2_ thin film is higher than that in the RP-HfO_2_ thin film. This is also consistent with the tendencies of lower breakdown voltages for DP-HfO_2_ thin films (Figure 1). Nevertheless, this result does not explain why the RP-HfO_2_ thin film, which has fewer defects that can act as charge-trapping sites, exhibits better ∆*V_FB_* characteristics than those of the DP-HfO_2_ thin film.

**Figure 4:**
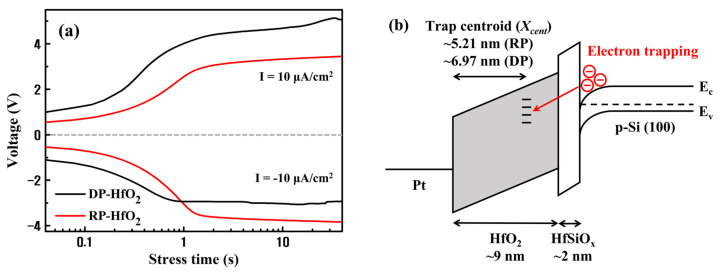
(**a**) Constant current stress measurements and (**b**) electron band diagrams including the trap centroids of DP- and RP-HfO_2_ MIS capacitors.

To determine the reason for the outstanding charge trapping properties of RP-HfO_2_ thin films, constant current stress (CCS) measurements were performed. CCS analysis is a widely applied method to estimate the charge trap centroid (*X_cent_*) of bilayer gate stacks [29,36,37]. These measurements allowed us to identify the majority trap sites in the HfO_2_ thin film. The capacitor was exposed to a constant current density of ±10 μA/cm^2^, and the shift in voltage was measured in the I-V characteristic as the stress time increased. The voltage shift was caused by the trapping of charges in the oxide layer. *X_cent_* of the capacitor was evaluated via the CCS measurement using the following equation:

(2) $$X_{cent}=\frac{t_{stack}}{\left[1-(∆V_{g}^{-}/∆V_{g}^{+})\right]}$$

where *X_cent_* is the distance from the gate electrode, *t_stack_* is the thickness of the oxide layer, and ∆*V_g_*^+^ and ∆*V_g_*^−^ are the positive and negative voltage shifts after the application of a CCS, respectively. TEM images of the cross-sections of DP- and RP-HfO_2_ MIS capacitors indicated that the thickness of the deposited HfO_2_ film was approximately 9 nm and an interfacial layer with a thickness of approximately 2 nm formed by an interfacial reaction existed between HfO_2_ and Si. Previous studies have demonstrated that the interfacial layers generated during the deposition of HfO_2_ thin films on Si wafers by DP and RPALD were Hf- and Si-rich Hf-silicates, respectively. This difference between the compositions of DP- and RP-HfO_2_ thin films is because of the interaction between Hf and SiO_2−x_ induced by energetic reactants in the plasma states [38,39]. Assuming that the dielectric constant of the deposited HfO_2_ film is comparable to the bulk value, the relative permittivity of the 2 nm thick Hf-silicate interfacial layer is estimated to be approximately 4.5. Figure 4a depicts the CCS measurement results for DP- and RP-HfO_2_ MIS capacitors. The *X_cent_* values calculated using Equation (2) for DP- and RP-HfO_2_ MIS capacitors were 6.97 and 5.21 nm, respectively. Figure 4b shows the structure and calculated *X_cent_* values of the HfO_2_ MIS capacitor along with the electronic band diagram. When a positive bias is applied to the gate electrode, the charge-trapping state in the HfO_2_ thin film is filled by the tunneling of the electrons accumulated on the Si surface. Considering the capacitance of HfO_2_ and HfSiO_x_ films, approximately 55% of the gate voltage can be allocated for electron tunneling. The presence of the majority of trap sites close to the interface in the DP-HfO_2_ film as compared to the case of the RP-HfO_2_ film implies that interface charge traps are prevalent as compared to bulk charge traps in DP-HfO_2_ films [29]. This result is believed to be caused by the formation of unstable interface defects and charge-trapping sites in the interfacial layer by plasma damage. This reduces the charge trapping efficiencies and ∆*V_FB_* values of DP-HfO_2_ MIS capacitors, and a larger voltage needs to be applied to the gate to compensate for these reductions [40,41].

**Figure 5:**
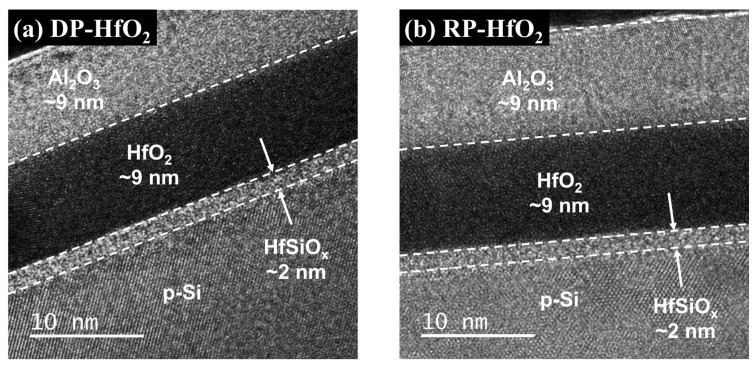
Cross-sectional transmission electron microscopy images of (**a**) DP- and (**b**) RP-HfO_2_ charge-trapping memory (CTM) devices.

After fabricating the CTM devices utilizing the DP- and RP-HfO_2_ thin films as CTLs (DP- and RP-HfO_2_ CTM devices, respectively), we evaluated the memory characteristics of these devices via electrical measurements. The Hf-silicate interfacial layer produced via the interfacial reaction between HfO_2_ and Si wafer was used as the TO to examine the effects of the plasma damage caused by DPALD on the formation of interface defects and the resulting changes in memory characteristics in the same way as for previous MIS capacitors. As the BO, 9 nm thick Al_2_O_3_ thin films were separately deposited by PEALD on the DP- and RP-HfO_2_ CTLs. After depositing a single Al_2_O_3_ thin film on a Si wafer, the deposited Al_2_O_3_ film was subjected to C–V measurements before its application to the CTM device to confirm the absence of charge trapping at the applied voltage. The deposited Al_2_O_3_ thin film exhibited an amorphous state, with its relative permittivity calculated from the C–V measurement approximately at 8.5. Figure 5a,b shows the cross-sectional TEM images of the DP- and RP-HfO_2_ CTM devices, respectively. In both devices, a Hf-silicate interfacial layer of approximately 2 nm was formed by a chemical reaction and atom mixing between HfO_2_ and Si. This interfacial layer is undesirable to achieve a small EOT for metal-oxide-silicon transistor applications in high-k dielectrics. However, it is suitable for application as a TO in CTM devices to suppress the detrapping of the trapped electrons or holes [5,42]. Both the DP- and RP- HfO_2_ thin films were mainly in an amorphous state.

**Figure 6:**
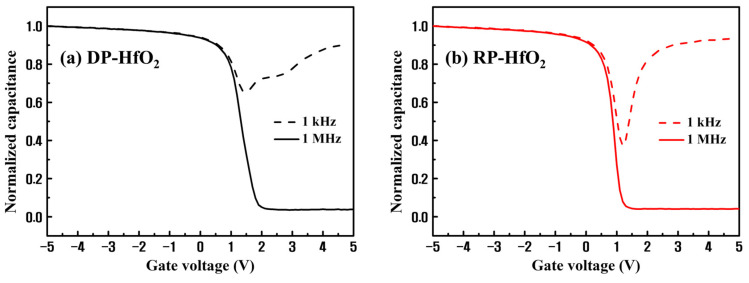
C–V measurement results of (**a**) DP- and (**b**) RP-HfO_2_ CTM devices at high (1 MHz) and low frequencies (1 kHz).

Figure 6 depicts the C–V measurement results at high (1 MHz) and low frequencies (1 kHz) for the DP- and RP-HfO_2_ CTM devices. Initially, the interface defects between HfO_2_ and Si extend the voltage direction of the curve. This indicates that an additional charge or voltage must be applied to fill the traps at the interface to achieve the same surface potential or band bending as that without the interface defects. The defects can be present at both interfaces of the HfSiO_x_ layer. Among them, the defects at the HfSiO_x_/Si interface have a more pronounced impact on the Si channel. Moreover, interface defects create a gap between the low- and high-frequency curves at the point V_min_ just before the occurrence of strong inversion [43,44]. This difference is proportional to the interface defect density *D_it_*; *D_it_* can be quantified by the high- and low-frequency capacitance method suggested by Castagné and Vapaille [45]:

(3) $$D_{it}=\frac{C_{ox}}{q^{2}}\left(\frac{C_{lf}/C_{ox}}{1-C_{lf}/C_{ox}}-\frac{C_{hf}/C_{ox}}{1-C_{hf}/C_{ox}}\right)$$

where *C_lf_* and *C_hf_* are the measured capacitances at low and high frequencies, respectively. The midgap *D_it_* values of the DP- and RP-HfO_2_ CTM devices evaluated using Equation (3) are 5.53 × 10^12^ and 1.18 × 10^12^ cm^−2^·eV^−1^, respectively. The *D_it_* value of the DP-HfO_2_ CTM device is approximately five times that of the RP-HfO_2_ CTM device, which is in appropriate agreement with the abovementioned I–V characteristics and CCS measurement results of the DP- and RP-HfO_2_ thin films.

**Figure 7:**
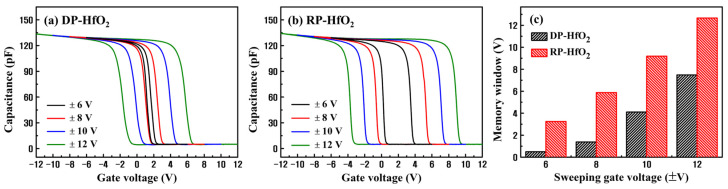
C–V characteristics of (**a**) DP- and (**b**) RP-HfO_2_ CTM devices as functions of sweeping voltage and (**c**) the corresponding memory windows.

Figure 7 shows a comparison of the variations of C–V characteristics and ∆*V_FB_* values of the DP- and RP-HfO_2_ CTM devices with respect to the sweeping voltage. The RP-HfO_2_ CTM device demonstrated wide ∆*V_FB_* values of 3.25 and 12.66 V at the operating voltages of ±6 and ±12 V, respectively. In contrast, the DP-HfO_2_ CTM device exhibited the ∆*V_FB_* values of 0.49 and 7.48 V at ±6 and ±12 V, respectively. ∆*V_FB_* of the RP-HfO_2_ CTM device demonstrated a high linearity in proportion to increasing sweeping voltage. This suggests that multiple levels of charge-storage nonvolatile memory can be implemented in this device [18].

Table 1 presents a comparison of the memory characteristics of previously reported high-k oxide-based CTM devices with those of the RP-HfO_2_ CTM devices fabricated herein. The CTM device synthesized in this study exhibits the highest ∆*V_FB_* characteristics even at lower annealing temperatures and lower drive voltages as compared to those of the previously reported devices.

**Figure 8:**
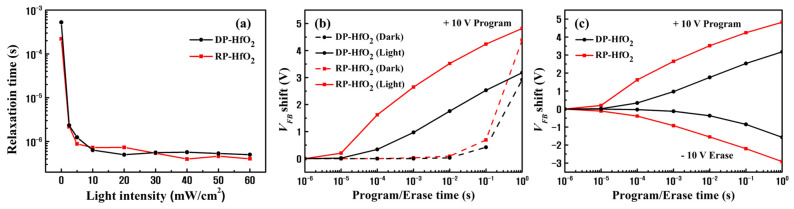
Comparison of the variations of the (**a**) relaxation time with respect to light intensity, (**b**) program speed with and without light irradiation, and (**c**) program/erase (P/E) rates for DP-HfO_2_ and RP-HfO_2_ CTM devices.

Applicabilities of the CTM devices to practical nonvolatile memory devices were determined via electrical measurements. The CTM capacitor structure demonstrates the ability to evaluate the electrical characteristics of the gate structure via a simple process and has a short fabrication time. Nevertheless, contrary to the transistor structure, which has a source and drain to facilitate the supply of minority carriers to the gate channel, the capacitor structure provides minority carriers to the channel via thermal generation. Therefore, the formation of the inversion layer is very slow. Consequently, the production and charging of minority carriers during programmable gate voltage application in an n-channel capacitor does not match the speed of the gate bias pulse. This can lead to inaccurate measurements of the P/E speed associated with the minority carriers [49]. To address this issue, we irradiated the device with light during the P/E speed measurement. The light irradiation source was a blue spectrum light-emitting diode lamp. During light irradiation, electron–hole pairs were generated around the capacitor, and the photogenerated electrons diffused into the channel region at the base of the gate structure, rapidly forming an inversion layer in response to the gate voltage pulse. Herein, the time required for the formation of the inversion layer follows the relaxation time *τ* of the dielectric constant model proposed by Debye. Using this model, an expression for the capacitance *C_inv_* in the inversion region as a function of frequency can be obtained as follows [50]:

(4) $$C_{inv}\left(\omega \right)=C_{hf}+\frac{C_{qe}-C_{hf}}{1+{\left(\omega \tau \right)}^{2}}$$

where *ω* is the angular frequency and *C_qe_* and *C_hf_* are the capacitances at quasi-static and high frequencies, respectively. Figure 8a shows the relaxation time as a function of light irradiance (P_a_) for the DP- and RP-HfO_2_ CTM devices. For both devices, *τ* decreased with an increase in P_a_ and saturated at approximately 0.5 μs. For accurate P/E measurements, P_a_ should be sufficiently large such that *τ* would reach the saturation value. Accordingly, the P/E speeds of the DP- and RP-HfO_2_ CTM devices were measured at P_a_ = 30 mW/cm^2^. The magnitude of the applied gate voltage was 10 V. Figure 8b depicts the variations of the program speeds of the DP- and RP-HfO_2_ CTM devices with and without light irradiation. The program speeds of the CTM devices evaluated under light irradiation were considerably higher than those measured in the dark room. Figure 8c shows the P/E speeds of the DP- and RP-HfO_2_ CTM devices under light irradiation. The RP-HfO_2_ CTM device demonstrated ∆*V_FB_* values of 2.01 and 5.07 V at the voltage application times of 10^−4^ and 10^−2^ s, respectively, due to its excellent charge trapping properties.

**Figure 9:**
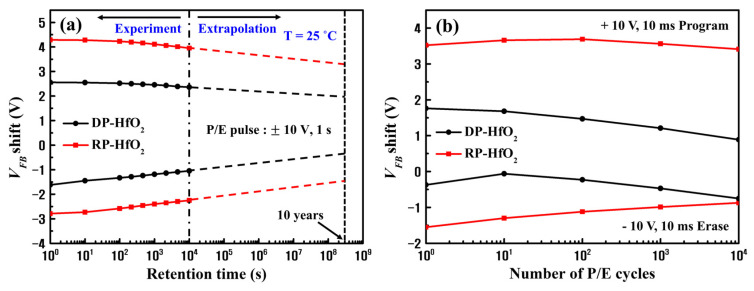
(**a**) Memory retention times and (**b**) endurance characteristics of DP- and RP-HfO_2_ CTM devices.

To evaluate the reliability characteristics of the fabricated DP and RP-HfO_2_ CTM devices, we measured the *V_FB_* shift as a function of memory retention time and P/E cycle. ∆*V_FB_* values of the synthesized CTM devices decreased with an increase in the memory retention time (Figure 9a). The voltage application conditions were set as follows: program pulse: 10 V for 1 s and erase pulse: −10 V for 1 s. The logarithmic of memory retention time is depicted in Figure 9a. The logarithmic behavior of the memory retention time was estimated, and the *V_FB_* shift for up to 10 years was extrapolated. At room temperature, the ∆*V_FB_* of the DP-HfO_2_ CTM device decreased by 46.89% over 10 years of memory retention as compared to that for the RP-HfO_2_ CTM device (34.32%). A larger charge loss occurred in the case of the DP-HfO_2_ CTM device due to interface defects between the Hf-silicate used as the TO and Si substrate and charge-trapping sites inside the TO, which easily detrapped the charges trapped inside the CTL [40,51]. However, even the RP-HfO_2_ CTM device demonstrated lower memory retention characteristics than those of previously reported high-k oxide-based CTM devices (Table 1). This is possibly caused by the relatively thin TO and BO as compared to those used in other devices. Further optimization of the structure is expected to enable the fabrication of devices with appropriate ∆*V_FB_* and memory retention characteristics. Figure 9b shows the *V_FB_* shift as a function of the number of P/E cycles for the DP- and RP-HfO_2_ CTM devices. P/E cycling was performed using a pulse train of ±10 V and 10 ms. For both DP- and RP-HfO_2_ CTM devices, the size of the ∆*V_FB_* slightly decreased during 10^4^ cycles. Nevertheless, for the DP-HfO_2_ CTM device, an overall shift of *V_FB_* in the negative direction was observed. Based on previous studies, this is believed to be owing to the generation of a fixed oxide charge inside the unstable Hf-silicate interfacial layer of the DP-HfO_2_ CTM device by the P/E cycling, overall decreasing the *V_FB_* [52]. This shift in *V_FB_* can cause a cycling-dependent decrease in the reliability of the device during real-world memory operation and should be maximally suppressed [53].

## 4. Conclusions

Herein, we determined the optimal process temperature for the DPALD and RPALD of HfO_2_ thin films and analyzed the influences of the plasma application method on the charge trapping properties of HfO_2_ thin films and properties of the interface between Si and HfO_2_ via various techniques. Subsequently, we fabricated CTM devices utilizing the deposited thin films as CTLs and evaluated their memory properties. The DP-HfO_2_ thin films exhibited relatively constant leakage current and breakdown voltage over the temperature range within the process window. However, the RP-HfO_2_ thin films demonstrated the lowest leakage current and highest breakdown voltage at an optimal process temperature within the process window. Thereafter, MIS capacitors were synthesized using the HfO_2_ thin films deposited at an optimal temperature of 220 °C, and C–V measurements were conducted. The results indicated higher ∆*V_FB_* characteristics of the RP-HfO_2_ MIS capacitors than those of the DP-HfO_2_ MIS capacitors. XPS depth profiling and CCS measurements were used to investigate the difference between the charge trapping properties of DP- and RP-HfO_2_ thin films; we discovered that the DP-HfO_2_ films contained many defects in the bulk and at the interface due to in-film damage caused by DP and an unstable interfacial reaction of HfO_2_ with Si. The memory characteristics of the RP-HfO_2_ CTM devices were excellent. Particularly, the ∆*V_FB_* of these devices was considerably large (±12.66 V) at an operating voltage of 12 V, suggesting that the RP-HfO_2_ CTM device may be suitable for future implementations of multiple levels of charge-storage nonvolatile memories. Moreover, the RP-HfO_2_ thin film shows promise for synaptic devices that demonstrate linear potentiation characteristics, provided an appropriate input pulse signal is utilized, owing to its high charge trapping density and low interface trap charge.

## Figures and Tables

**Table 1:** Comparison among the memory characteristics of high-k-based CTM devices with different structures and compositions.

TO/CTL/BO	Thickness (nm)	Annealing Temp. (°C)	Operating Voltage (V)	Memory Window (V)	Charge Loss (%)	References
HfSiO_X_/RP-HfO_2_/Al_2_O_3_	2/9/9	400	±12	12.66	34.32	This work
SiO_2_/HfO_2_/Al_2_O_3_	3/10/10	1000	±15	7.4	31	[18]
SiO_2_/HfO_2_	3/55/0	800	±10	5.1	-	[5]
Al_2_O_3_/HfAlO/Al_2_O_3_	2/9/12	600	±12	6.29	79	[46]
Al_2_O_3_/HfAlO/Al_2_O_3_	2/10/15	450	±14	7.45	23.64	[47]
SiO_2_/ZrO_2_/Al_2_O_3_	5/10/15	700	±11	7.1	16	[9]
SiO_2_/HfAlO/Al_2_O_3_	3/9/8	800	±16	11.5	14.9	[48]
SiO_2_/Al-rich Al_2_O_3_/Al_2_O_3_	3.4/5/6	400	±12	8.2	-	[6]
(Al_2_O_3_/SiO_2_)/Ge/Al_2_O_3_	(4/3)/15/10	700	−1~14	5.41	11 (ON), 9.8 (OFF)	[16]

## Data Availability

The data presented in this study are contained within the article.
